# Isolation, characterization and identification of antigenotoxic and anticancerous indigenous probiotics and their prophylactic potential in experimental colon carcinogenesis

**DOI:** 10.1038/s41598-019-51361-z

**Published:** 2019-10-14

**Authors:** Deepika Chandel, Mridul Sharma, Vibhindika Chawla, Naresh Sachdeva, Geeta Shukla

**Affiliations:** 1 Department of Microbiology, Panjab University, Chandigarh, 160014 India; 2 Department of Endocrinology, Post Graduate Institute of Medical Education and Research, Chandigarh, 160012 India

**Keywords:** Cancer prevention, Applied microbiology

## Abstract

Colorectal cancer, the third most commonly diagnosed cancer, is a lifestyle disease where diet and gut microbiome contribute intricately in its initiation and progression. Prophylactic bio-interventions mainly probiotics offer an alternate approach towards reducing or delaying its progression. Therefore, the present study was designed wherein a robust protocol for the isolation, characterization, and identification of indigenous probiotics having antigenotoxic and anticancerous activity was followed along with their prophylactic potential assessment in early experimental colorectal carcinogenesis. Among forty-six isolated lactic acid bacterial strains, only three were selected on the basis of antigenotoxicity against N,N-Dimethyl dihydrazine dihydrochloride and 4-Nitroquinoline 1-oxide and probiotic attributes. All three selected probiotic strains exhibited anticancerous potential as is evident by the reduced Aberrant Crypt Foci, reduced fecal pH, enhanced fecal lactic acid bacteria and altered fecal enzymes (β-glucuronidase, nitroreductase, β-glucosidase) that modulated gut microbiota and microenvironment resulting into restored histoarchitecture of the colon. The results are a clear indicator of the prophylactic potential of selected indigenous probiotics which may be used as an alternative prophylactic biological therapy against colon carcinogenesis particularly in highly susceptible individuals.

## Introduction

Cancer, a heterogeneous disease, is characterized by uncontrolled growth and spread of abnormal cells. Cancer of colon and rectum are collectively known as colorectal cancer (CRC) and ranks third in incidence and second in mortality worldwide^1^. The prevalence of CRC is high in western countries and is rapidly increasing even in developed Asian countries^2^. There are various risk factors associated with CRC, including external factors, such as poor diet, alcohol and tobacco use and physical inactivity, and internal factors, such as hereditary genetic predispositions, hormone, and immunological imbalances^3^. The negative regulators of CRC may act in tandem or sequentially to either initiate cancer growth or to drive cancer development. Colon cancer arises through a sequential process where molecular and cellular modifications result in certain precursor lesions, i.e, the adenomatous polyps which can lead in to adenoma and ultimately into carcinoma. Conversion of normal mucosa to adenocarcinoma is a long process with several intermediating steps which offer scope for various prophylactic biointerventions such as probiotics the ‘live beneficial microorganisms’ to act as preventive agents and reduce the occurrence and burden of the disease^4,5^. The beneficial effects of probiotics include maintaining gut homeostasis, prevention of acute diarrhea, irritable bowel syndrome, colitis and constipation among others^6^. Several *in vitro*^7,8^, animal studies^9–12^ as well as clinical studies^13,14^ have indicated that probiotics have anticancerous attributes specifically against CRC^15–18^. Probiotics exert their anticancer activity mainly by modulation of gut microbiota, improvement of physico-chemical conditions of the colon, enhancing gut barrier function, modulating gut bacterial metabolism and enzymes thereby preventing from carcinogens, secreting anticancerous metabolites and reducing inflammation^19^. Enormously intricate interaction exists between probiotics and immune system which is difficult to evaluate but, the current experimental data gives a clear indication of the modulatory effect of LAB on colonic inflammation, mainly by the restoration of epithelial tight junctions and modulation of T-regulatory cells and their dendritic cells^14,18,20,21^.

Health promoting attributes associated with probiotics are enormously strain and species dependent^22^ and their prophylactic potential against colon cancer may also vary from one strain to another and warrants investigation. Therefore, the present study aimed at isolating LAB specifically targeted against experimental CRC having antigenotoxic and anticancerous potential.

## Results

### Isolation of Lactic acid bacteria (LAB)

**Figure 1:**
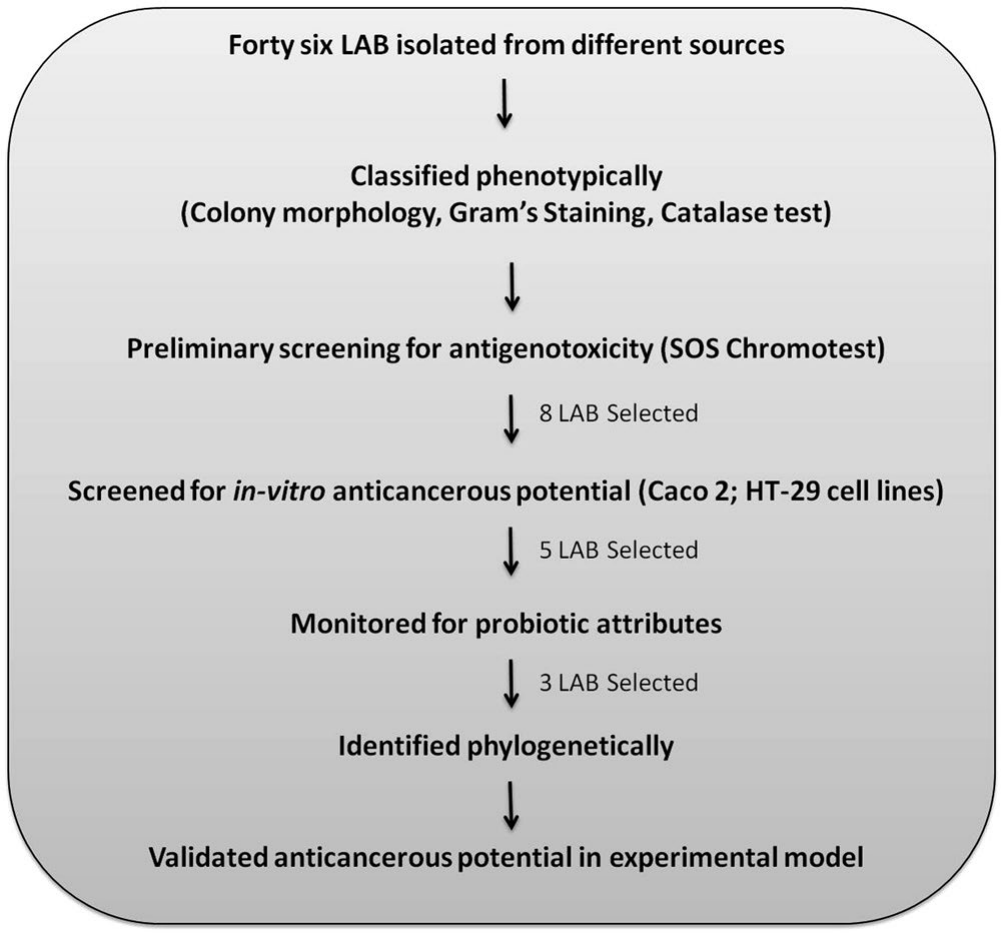
Flow diagram depicting the work flow for isolation, screening, characterization and validation of anticancerous probiotic strains.

Forty-six lactic acid bacterial isolates were isolated from different sources. Among these twenty-seven were from infant feces (#1–27) and nineteen from fruit peels (# 1A-19A). Most of these LAB showed white, circular, slightly elevated mucoid colony with a smooth texture on MRS agar and were either gram-positive rod or cocci and catalase negative (Data not shown). The isolates were then screened *in-vitro* for antigenotoxicity, anticancerous activity, monitored for probiotic attributes and selected isolates were identified and their anticancer potential was validated in experimental model of colon carcinogenesis (Fig. 1).

### Preliminary screening of isolated LAB for antigenotoxicity

**Figure 2:**
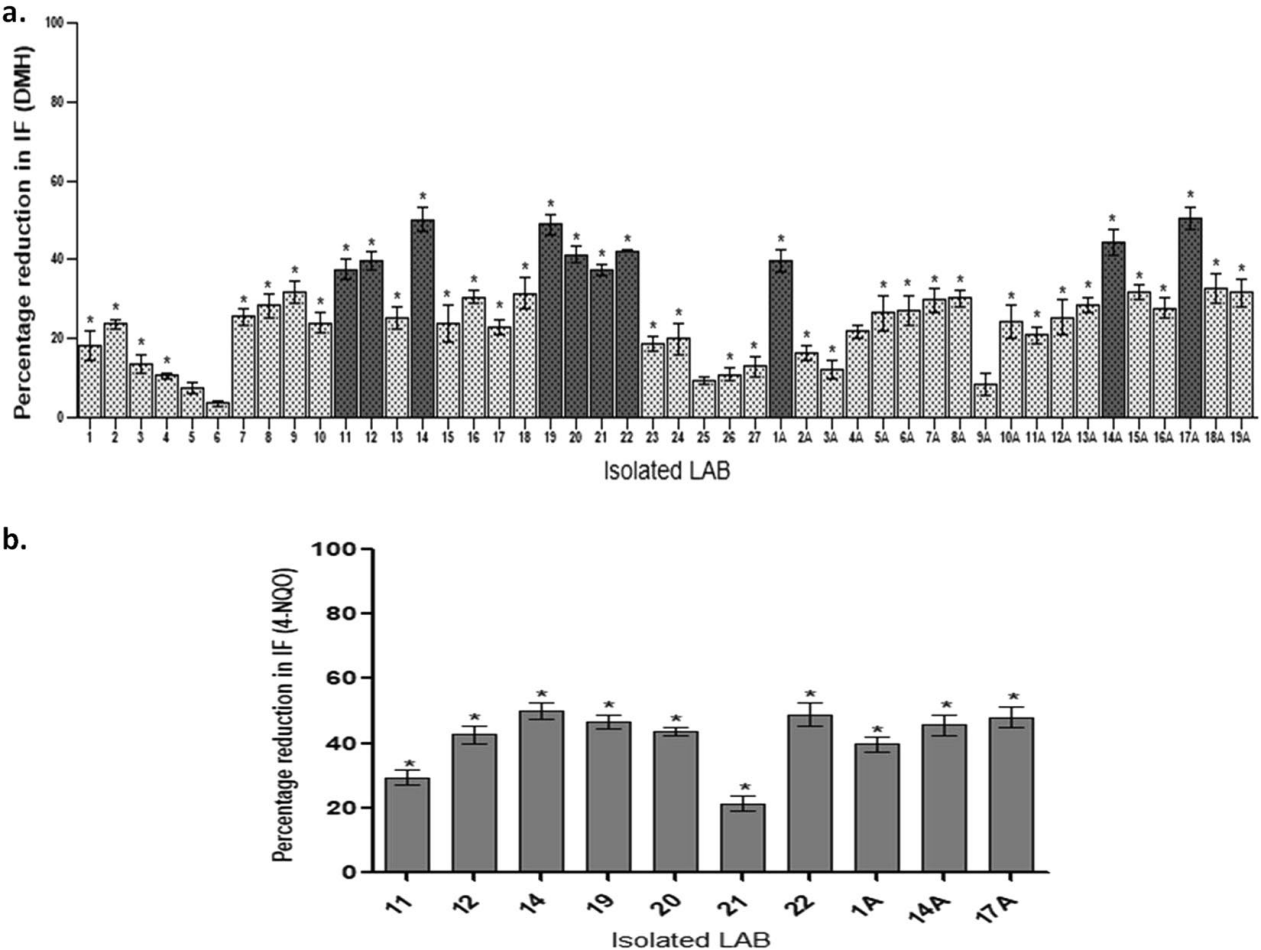
Antigenotoxicity (% reduction in induction factor) of isolated LAB against *E. coli* PQ37 induced by DMH (**a**); 4-NQO (**b**). Values are mean ± SD. *p < 0.05 v/s DMH/4-NQO.

It was found that seventeen isolates amongst forty-six isolates showed more than 30 percent reduction in the genotoxicity of N,N-Dimethyl dihydrazine dihydrochloride (DMH) (Fig. 2a). However, only ten isolates showed more than 40% reduction in the genotoxicity of DMH and were again screened for antigenotoxicity against 4-Nitroquinoline 1-oxide (4-NQO). Interestingly, it was found that only eight LAB isolates (#14, 12, 19, 20, 22, 1 A, 17 A, 14 A) out of ten exhibited more than thirty percent reduction in the genotoxicity of 4-NQO (Fig. 2b). Further, these eight isolates were selected to assess their anticancerous activity *in vitro* i.e. secondary screening.

### Secondary screening of isolated LAB for anticancerous activity *in vitro*

**Figure 3:**
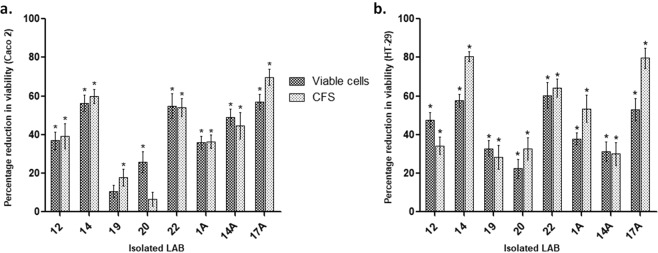
Effect of isolated LAB and their supernatants on the viability (percent reduction) of HT-29 (**a**) and Caco 2 cells (**b**). Values are mean ± SD *p < 0.01, #p < 0.05 v/s control.

To observe the anticancerous effect of isolated LAB *in vitro*, Caco 2 and HT-29 cells were employed. Isolated LAB viable cell culture as well as their cell-free supernatants (CFS) inhibited the proliferation of both Caco 2 and HT-29 cells. Specifically, antiproliferative potential against Caco 2 cells was most significant (p < 0.01) with LAB isolate #14 (56%) followed by LAB isolate #17A, 22, 14A, 12, 1A, 20 and 19 respectively (Fig. 3a). Although, it was also observed that CFS of all selected isolated LAB reduced the proliferation of Caco 2 cells but CFS of LAB isolate #17A had maximum reduction in proliferation of Caco 2 cells (69%) followed by CFS of LAB isolate #14, 22, 14A 12, 1A, 19 and 20 respectively (Fig. 3a). Thus it was observed that most of the selected isolated LAB cultures and their supernatants had antiproliferative effect on Caco-2 cells but viable cells of isolated LAB #14 had a maximum reduction in proliferation whereas CFS of isolated LAB #17A exhibited maximum antiproliferative activity.

Similarly, with HT-29 cells, all selected isolated LAB viable cell cultures and their CFS significantly (p < 0.01) reduced proliferation (Fig. 3b). It was observed that viable cells of LAB isolate #22 showed a marked reduction in the viability of HT 29 cells (60%) followed by LAB isolate #14, 17A, 12, 1A, 19, 14A and 20 respectively. Though, cell-free supernatants of the isolated LAB exhibited significant (p < 0.01) antiproliferative activity but maximum inhibition was observed with CFS of LAB isolate 14 followed by 17A, 22, 1A, 12, 20, 14A and 19 respectively.

Comparative analysis of both viable LAB cultures and CFS of selected isolated LAB showed anticancerous potential as it inhibited proliferation of both cell lines, therefore, five isolates exhibiting maximum antiproliferative effect were then selected to assess their probiotic properties.

### Probiotic characterization of selected LAB

Only five isolated LAB (#14, 20, 22, 17A and 1A) were selected based on antigenotoxic and anticancerous potential. Therefore, these were assessed for their probiotic attributes mainly acid and bile tolerance and cell surface hydrophobicity.

### Acid and bile tolerance

The ability of isolated LAB to survive in acidic condition (pH 2.0, 3.0) and bile salt concentration (0.2%, 0.4%) was evaluated to determine the survival of isolates under gastrointestinal conditions. Interestingly, it was observed that all selected isolated LAB tolerated acidic pH (2 and 3) and bile salts (0.2% and 0.4%) at each point of observation (Table 1).

**Table 1:** Probiotic attributes of selected isolated LAB.

Isolate	Acid Tolerance						Bile Tolerance				CSH	
	pH 2 log_10_ CFU mL^−1^			pH 3 log_10_ CFU mL^−1^			0.2% bile salt log_10_ CFU mL^−1^		0.4% bile salt log_10_ CFU mL^−1^		Hydrophobicity Percentage	
	0 h	1 h	2 h	0 h	1 h	2 h	2 h	4 h	2 h	4 h	Xylene	Hexadecane
14	7.2 ± 0.03	6.06 ± 0.02	3.08 ± 0.04	8.17 ± 0.04	7.32 ± 0.01	6.44 ± 0.03	8.1 ± 0.02	8.9 ± 0.3	7.87 ± 0.04	7.9 ± 0.4	22.26 ± 1.89	51.76 ± 3.6
20	7.9 ± 0.03	4.92 ± 0.02	2.08 ± 0.05	8.17 ± 0.03	7.04 ± 0.04	6.52 ± 0.03	7.18 ± 0.3	7.8 ± 0.02	7.17 ± 0.07	6.9 ± 0.09	51.9 ± 4.5	16.9 ± 2.1
22	7.15 ± 0.03	6.02 ± 0.01	4.83 ± 0.02	8.07 ± 0.04	7.58 ± 0.04	6.78 ± 0.04	7.46 ± 0.1	7.78 ± 0.3	7.29 ± 0.04	7.37 ± 0.4	29.47 ± 2.97	45.78 ± 3.1
1A	7.89 ± 0.04	7 ± 0.03	5.61 ± 0.04	8.21 ± 0.02	7.45 ± 0.04	6.3 ± 0.03	7.04 ± 0.4	8 ± 0.03	6.51 ± 0.02	6.7 ± 0.6	18.32 ± 2.7	41.08 ± 1.98
17A	7.94 ± 0.02	6.9 ± 0.05	3.77 ± 0.04	8.01 ± 0.03	7.12 ± 0.03	6.14 ± 0.04	7.25 ± 0.02	7.88 ± 0.3	7.01 ± 0.03	6.91 ± 0.3	42.75 ± 1.00	48.55 ± 3.02

Values are expressed as mean ± SD. CSH: Cell surface hydrophobicity.

### Cell surface hydrophobicity (CSH)

To assess the adhering ability of the selected isolated LAB, cell surface hydrophobicity was assessed using hydrocarbons (Xylene and Hexadecane). It was observed that with Xylene, LAB isolate 1A exhibited maximum hydrophobicity (28%) followed by LAB isolate 14 (22%), 20 (21%), 22 (21%) and 17A (18%) respectively. Further, with hexadecane maximum hydrophobicity of 41% was observed with isolate #14 followed by 17A (38%), 22 (35%), 1A (31%) and 20 (16%) respectively (Table 1).

### Identification of selected isolates

Only three Isolated LAB (14, 22 and 17A) were selected, based on anti-genotoxicity, anticancerous activity, probiotic characters and were further identified by 16s rRNA sequencing for species-level identification of bacteria. Sequences obtained were submitted to the GenBank. LAB Isolate 14 was identified as *Lactobacillus rhamnosus* (99% similarity), Genbank sequence ID MH656799. LAB Isolate 22 was identified as *Lactobacillus plantarum* (99% similarity), Genbank sequence ID MH656803 and LAB isolate 17A was found to be *Pediococcus pentosaceus* (99% similarity) Genbank sequence ID MH889142.

### *In vivo* study

The selected and identified isolated probiotics i.e. *L. rhamnosus* MD14, *L. plantarum* GMD and *P. pentosaceus* GMD17A were employed to assess their anticancerous potential in early experimental colon carcinogenesis.

### Body mass and growth rate

**Figure 4:**
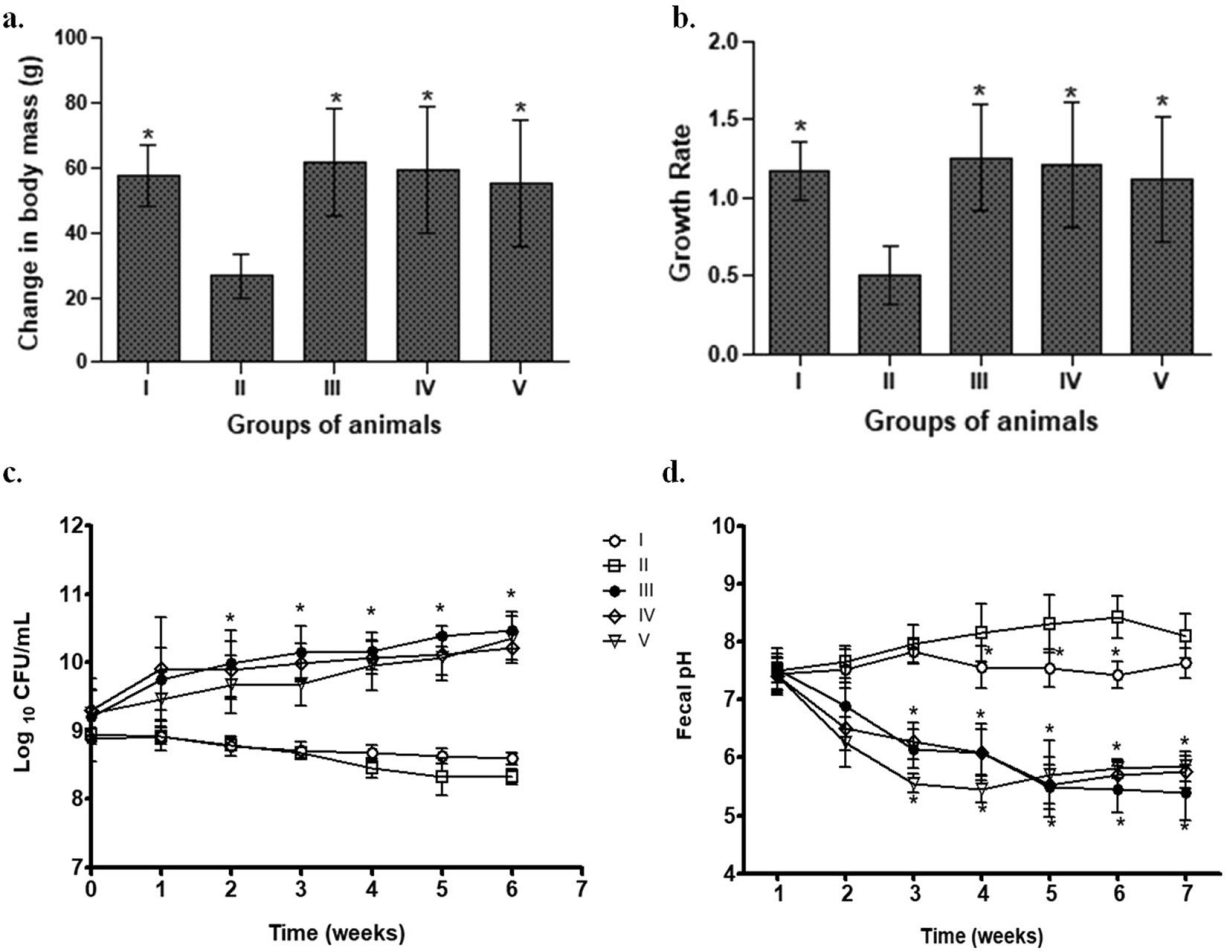
Effect of selected isolated LAB supplementation on change in body mass (**a**); growth rate (**b**); fecal pH (**c**); fecal lactic acid bacterial count (**d**) of animals belonging to different groups. Values are Mean ± SD, *p < 0.05 versus DMH-treated. Control (I); DMH-treated (II); *L. rhamnosus* MD14+ DMH-treated (III); *L. plantarum* GMD+ DMH-treated (IV); *P. pentosaceus* GMD17A+ DMH-treated (V).

It was observed that one-week prior supplementation of isolated probiotics before the initiation of DMH induced CRC, led to a gradual increase in body mass of animals belonging to all probiotic-fed animals (Fig. 4a). More specifically, it was observed that animals belonging to *L. rhamnosus* MD14+ DMH-treated (Group III) showed significant (p < 0.05) increase in both body mass and growth rate followed by *L. plantarum* GMD+ DMH-treated (Group IV) and *P. pentosaceus* GMD17A+ DMH-treated (Group V) compared with DMH-treated animals (Group II; Fig. 4a,b), which had least increase in body mass and lowest growth rate.

### Fecal lactobacilli count

The recovery of lactobacilli in feces indicates the survival, colonizing and transiting ability of probiotics thereby modifying the gut microbiota and microenvironment. It was observed that though prior supplementation of different probiotics led to a gradual increase in lactobacilli count in all probiotic-fed animals but *L. rhamnosus* MD14+ DMH-treated (Group III) had significantly (p < 0.05) higher lactobacilli count, followed by *L. plantarum* GMD+ DMH-treated (Group IV), *P. pentosaceus* GMD17A+ DMH-treated (Group V) compared with DMH-treated (Group II) animals (Fig. 4c).

### Fecal pH

It has been hypothesized that alkaline feces increases the risk of colon cancer therefore, the fecal pH of animals belonging to different groups was assessed weekly. It was observed that fecal pH was comparable among animals belonging to all the groups with no significant difference at the beginning of experiment. However, fecal acidification was observed from the second week onwards with probiotic supplementation. It was interesting to observe that at the end of experiment, animals belonging to *L. rhamnosus* MD14+ DMH-treated (Group III) had maximum acidification of feces (pH-5.4), followed by *L. plantarum* GMD+ DMH-treated (Group IV; pH-5.8) and *P. pentosaceus* GMD17A+ DMH-treated (Group V; pH-5.9) compared with DMH-treated animals (Group II; pH 8.1) and control animals (Group I; pH 7.6; Fig. 4d).

### Liver function test

**Figure 5:**
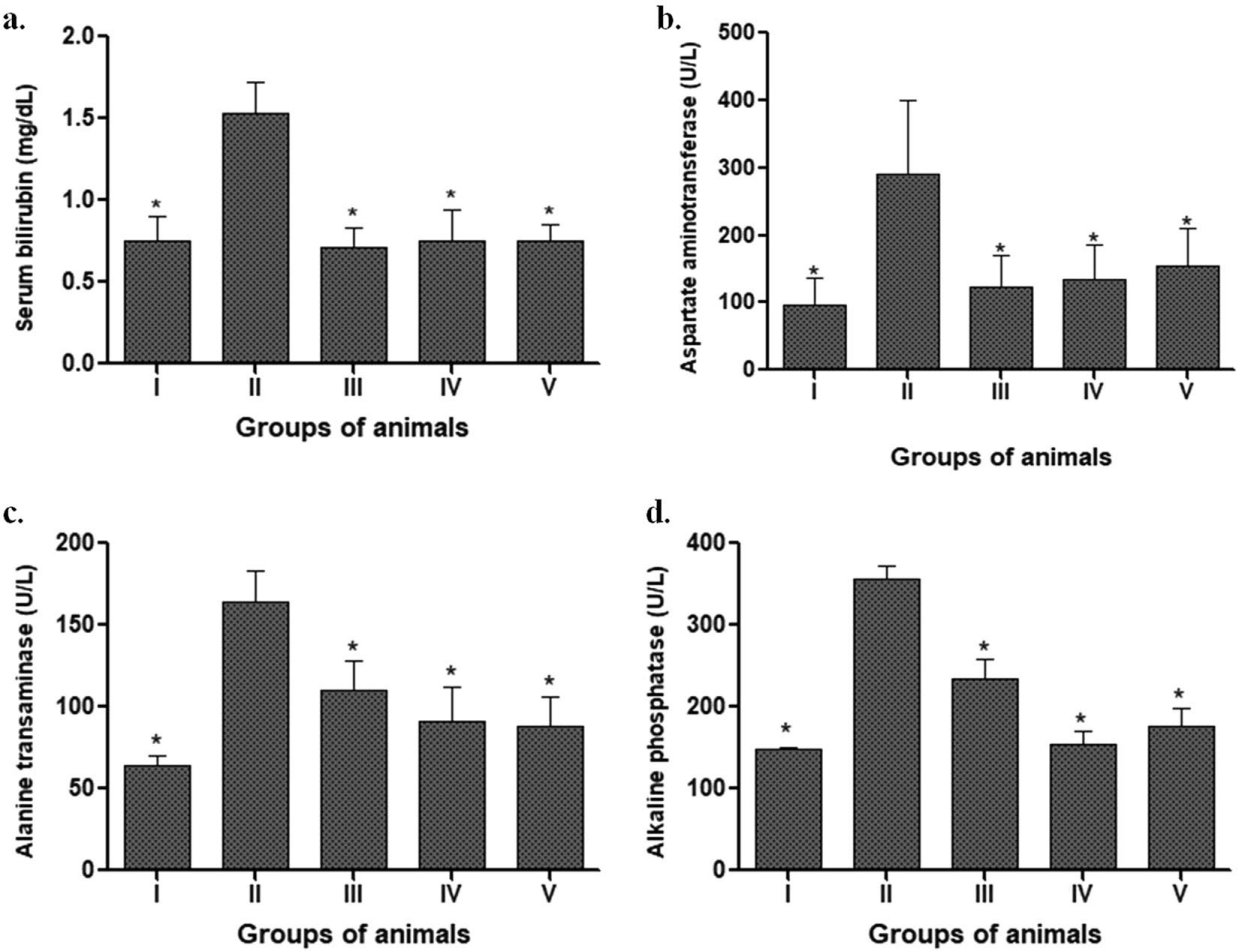
Effect of selected isolated LAB supplementation on the liver function: Serum bilirubin (**a**); Aspartate aminotransferase (AST, **b**); Alanine transaminase (ALT, **c**); Alkaline phosphatase (AP, **d**) in animals belonging to various groups. Values are Mean ± SD, *p < 0.05 versus DMH-treated. Control (I); DMH-treated (II); *L. rhamnosus* MD14+ DMH-treated (III); *L. plantarum* GMD+ DMH-treated (IV); *P. pentosaceus* GMD17A+ DMH-treated (V).

The plasma levels of serum bilirubin, ALT, AST and Alkaline phosphatase (AP) were measured as an indicator of liver function in order to assess the effect of LAB supplementation on DMH-treated animals. More specifically, it was observed that animals fed with different isolated probiotic led to a significant decrease (p < 0.05) in all the liver biomarkers in spite of DMH treatment compared with DMH-treated animals (Group II) that had elevated levels of serum bilirubin, ALT, AST and AP (Fig. 5).

### Aberrant crypt foci

Aberrant crypt foci (ACF), the earliest and reliable hallmark of colon carcinogenesis at the early stage of experimental carcinogenesis, were found to be significantly decreased (p < 0.05) in all probiotic treated animals compared with DMH-treated animals (Supplementary Table 1). Interestingly, it was observed that animals belonging to *L. rhamnosus* MD14+ DMH-treated (Group III) had significantly (p < 0.05) high percentage of ACF reduction (79.73%) followed by *L. plantarum* GMD+ DMH-treated (Group IV; 64.05%) and *P. pentosaceus* GMD17A+ DMH-treated (Group V; 57.5%).

### Fecal enzymes assay

**Figure 6:**
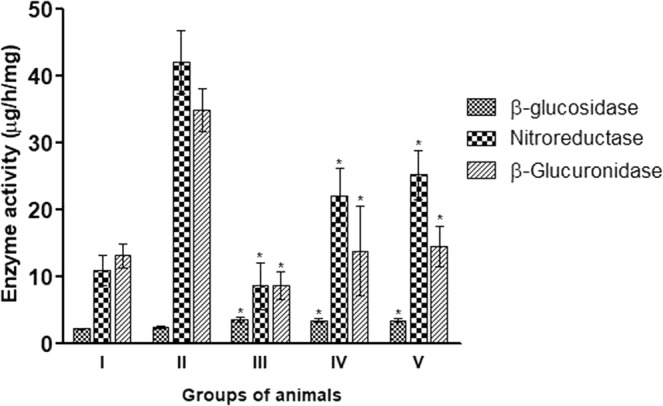
Effect of selected isolated LAB supplementation on fecal bacterial enzyme activities (µg/h/mg) of animals belonging to various groups. Values are Mean ± SD, *p < 0.05 versus DMH-treated. Control (I); DMH-treated (II); *L. rhamnosus* MD14+ DMH-treated (III); *L. plantarum* GMD+ DMH-treated (IV); *P. pentosaceus* GMD17A+ DMH-treated (V).

Fecal enzymes i.e. β-glucuronidase, nitroreductase, β-glucosidase have been implicated in converting pro-carcinogens into carcinogens thus the activity of these enzymes were assessed to deduce the modulating potential of isolated probiotic in the colonic environment. It was observed that animals supplemented with probiotics along with DMH-treatment had reduced β-glucuronidase and nitroreductase activity with increased β-glucosidase activity (Fig. 6). Specifically, it was observed that animals belonging to *L. rhamnosus* MD14+ DMH-treated (Group III) had significant (p < 0.05) reduction in the activity of β-glucuronidase and nitroreductase and increased activity of β-glucosidase followed by *P. pentosaceus* GMD17A+ DMH-treated (Group V) and *L. plantarum* GMD+ DMH-treated (Group IV) compared with DMH-treated animals (Fig. 6).

### Histopathological study

**Figure 7:**
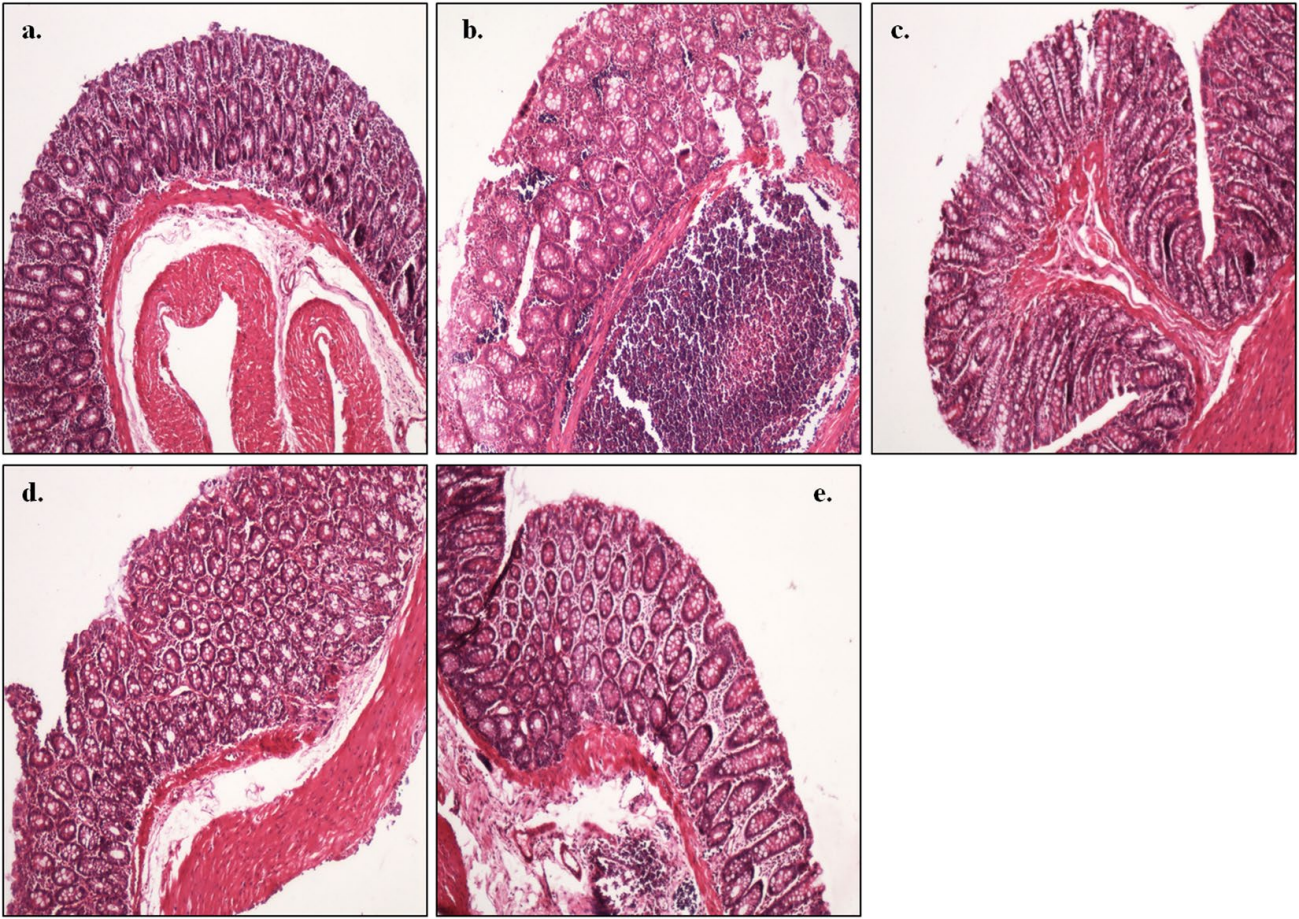
Representative photomicrograph of the colon of animals showing: (**a**) normal colon morphology in control animals; (**b**) disrupted crypts and dysplasia in DMH-treated animals; (**c**) closely packed mucus glands with minimal inflammatory infiltrate in *L. rhamnosus* MD14+ DMH-treated animals; (**d**) moderate inflammatory infiltrate in *L. plantarum* GMD+ DMH-treated animals; (**e**) dense inflammatory infiltrate and edema in *P. pentosaceus* GMD17A+ DMH-treated animals (H&E staining, 100X).

Histology of the colon of control animals (Group I) exhibited normal histo-architecture and cellular morphology of mucosa, submucosa, and muscularis propria with intact epithelium lining (Fig. 7a) compared with disrupted crypts and mucous glands with moderate degree of dysplasia in the form of glandular dilation along with markedly dense inflammatory infiltrate comprising of inflammatory cells, lymphocytes and plasma cells forming a lymphoid nodule along with pseudostratification and hyperchromasia in DMH-treated (Group II) animals (Fig. 7b). Interestingly, the colon of *L. rhamnosus* MD14+ DMH-treated (Group III) showed intact epithelium lining and closely packed mucus glands with minimal inflammatory infiltrate comprising of eosinophil, plasma cells, neutrophils (Fig. 7c) compared with moderate inflammatory infiltrate forming lymphoid nodule in the submucosal layer in *L. plantarum* GMD+ DMH-treated (Group IV) animals (Fig. 7d). Similarly, the colonic tissue of animals supplemented with *P. pentosaceus* GMD17A+ DMH-treated (Group V) had dense inflammatory cells along with edema in lamina propria (Fig. 7e).

## Discussion

Colorectal cancer, a lifestyle disease, remains a leading cause of morbidity and mortality worldwide. Among other factors such as genetics, age, exposure to carcinogens, lifestyle, smoking, and alcohol use, diet plays an important contribution to its health risk^3^. Ample evidence suggests that colonic microflora is greatly involved in the etiology of CRC, therefore, dietary interventions and natural bioactive supplements such as probiotics have been studied experimentally extensively to reduce the risk of CRC^13,17,20^. In our earlier studies, we found that prior supplementation of either established probiotics (*L. rhamnosus* GG, *L. acidophilus*) alone or in combination with Celecoxib exhibited anti-cancerous activity as evident by reduced ACF and procarcinogenic biomarkers: NFκB, COX-2, β-catenin, K-ras^9–12^. Since the response of probiotics is species and strain specific, the present study was aimed at isolating indigenous probiotics exhibiting antigenotoxic and anticancerous potential vis-à-vis assessing prophylactic potential in experimental CRC.

We observed that isolated LAB from infant fecal samples and fruit peels possessed antigenotoxic and anticancerous activities as these are regarded as the functional properties for characterizing probiotic microorganisms to be used either as prophylactic agents or to reduce the occurrence of CRC in highly susceptible individuals^21,23,24^. In earlier studies, it has been reported that LAB and bifidobacteria inhibited the genotoxic effect of 4-NQO in SOS-Chromotest^23,25,26^. Interestingly, in present study as well, it was observed that both isolated LAB cultures and their respective CFS inhibited the growth of Caco-2 and HT-29 cell lines indicative of antiproliferative potential and corroborates with earlier studies which have also reported that probiotics, both live cultures, and heat-killed cells, and their respective metabolites inhibit the growth of HT-29, HeLa Caco-2 and PANC-1cancer cell lines^7,24,27^.

To further validate the anti-cancerous potential of selected indigenous probiotics, DMH model of experimental colon carcinogenesis was employed as it is a well established, preferred and most widely used model that mimics human sporadic CRC^28^. It was observed that prior supplementation of selected indigenous probiotic resulted in a gradual increase in body mass and growth rate in spite of DMH treatment and is in accordance with earlier^9–12,15,16^. In these studies, it was observed that prior supplementation of *L. acidophilus* and *L. rhamnosus* GG alone or in combination with celecoxib (NSAID) led to an increase in both body mass and growth rate of animals.

Interestingly, in the present study, we observed that continued supplementation of indigenous probiotics, prior to initiation of CRC with DMH, enhanced the fecal lactic acid bacterial count indicating the survival and transiting ability of probiotics thereby modifying the gut microbiota and microenvironment and corroborates with earlier observations^12,15^. Epidemiologically, it has been shown that populations with alkaline fecal pH are at greater risk for colon cancer than populations with acid fecal pH^29^. The observed acidification of fecal pH in the present study may be due to the generation of Short Chain Fatty Acids (butyrate and conjugated linoleic acids) as scientists have also observed that acidification of feces occurred due to probiotic *L. acidophilus* administration in a DMH induced carcinogenic rat model^30^.

The prophylactic potential of indigenous probiotics was also observed by assessing the hepatophysiology, as the liver is the pivotal organ playing an important role in the normal physiology of human beings. A remarkable reduction in the liver biomarkers (ALT, AST, Alkaline phosphatase and Serum Bilirubin) were observed in probiotic-supplemented animals despite DMH treatment compared with DMH treated animals, where severe liver damage was observed. The results are suggestive of attenuation of cellular leakage and restoration of functional integrity of cell membrane in the liver due to probiotic supplementation. Earlier reports have also documented that prior administration of different probiotics (Lactobacilli and *Bifidobacterium)* in an acute liver injury rat model reduced hepatocellular damage as well as attenuated liver injury as observed by ALT, bilirubin, and glutathione levels^31,32^.

Aberrant crypt foci (ACF), the earliest preneoplastic marker of CRC, were found to be reduced in animals administered with probiotics. However, the percentage reduction in ACF was variable among probiotics but is comparable with the observation of Verma and Shukla^12^. They have also reported that different probiotics have different percentage of ACF reduction and was maximum with *L.GG* followed by *L. acidophilus* and *L. plantarum* treated animals respectively. The possible mechanism of reduced ACF in probiotics + DMH treated animals may be due to the difference in the interaction of isolated LAB with DMH metabolites that may have altered the intestinal microenvironment mainly by alteration of gut pH and metabolites produced, thus preventing DNA damage in the colon. Moreover, the observed different percentage of ACF reduction by different probiotics may also be due to the fact that probiotic response is very much species and strain specific^22^.

Fecal bacterial enzyme activities were monitored to assess the effect of selected probiotics on the colonic microenvironment as these are implicated in converting pro-carcinogens into carcinogens. It was observed that prior supplementation of selected indigenous probiotic led to decreased activity of β-glucuronidase and nitroreductase suggesting their role in preventing chemical carcinogenesis as β-glucuronidase is responsible for converting DMH to its ultimate carcinogen, methylazoxymethanol^33^. All selected probiotic-supplemented animals had high levels of β-glucosidase activity which may be due to the higher β-glucosidase activity in lactobacilli as it is implemented in carbohydrate metabolism through the catabolism of cellobiose and other β-glucosides^34^. The prophylactic potential of selected probiotics was also validated by in the histopathological observations where normal histoarchitecture of colons of probiotic-supplemented animals was observed. Moreover, lymphocytic infiltration was found to be reduced along with normal mucus glands in probiotic-supplemented animals despite DMH treatment and corroborates with our earlier observations^9–12^.

Taking into consideration the findings of the present study, it can be stated that probiotics confer protection against CRC mainly by improving homeostasis in the colon which lowers fecal pH and modulates metabolism by altering preneoplastic fecal enzymes resulting in the elimination of toxins and carcinogens. Additionally, probiotics ameliorate liver physiology *vis-a-vis* attenuate colonic damage. However, a further detailed study is underway to assess the role of selected indigenous probiotic in the modulation of various carcinogenic molecular markers in experimental colon carcinogenesis.

## Materials and Methods

### Materials

Chemicals including Minimum Essential Medium Eagle (MEM), Roswell Park Memorial Institute Medium (RPMI), penicillin, ampicillin, streptomycin, Fetal Bovine Serum (FBS), O-Nitrophenyl-β-D-galactopyranoside (ONPG), p-Nitrophenyl phosphate disodium salt hexahydrate (PNPP), Histidine, De Man Rogosa and Sharpe (MRS) media, Glycerol, MTT (3-(4,5-Dimethylthiazol-2-yl)-2,5-Diphenyltetrazolium Bromide were purchased from Hi-media, Mumbai, India. 4-Nitroquinoline *N*-oxide (4-NQO), Hexadecane, N,N-Dimethyl dihydrazine dihydrochloride (DMH), Dimethyl sulfoxide (DMSO) were purchased from Sigma-Aldrich (Merck) India. Universal primers (UNI 27F-5′ AGAGTTTGATCCTGGCTGAG 3′, UNI 1492R-5′ GGTTACCTTGTTACGACTT 3′) were procured from Eurofins, India.

### Isolation of lactic acid bacteria (LAB)

Lactic acid bacteria (LAB) were isolated from fruit peels (apple, orange) and fresh neonatal fecal samples from human infants collected in sterile containers. A total of 20 fecal samples from healthy breastfed infants (1–6-month-old) were collected only after obtaining informed consent from the parents in a congenial environment. Briefly, fecal sample (1 g) was serially diluted and inoculated on MRS agar, incubated at 37 °C for 48 h, 3 to 5 single colonies of each sample with different morphologies were randomly selected and again inoculated to MRS broth and streaked on MRS agar several times to get pure culture. The pure isolates were Gram stained for the preliminary identification. Purified cultures were maintained in MRS broth for daily use and preserved in 50% (v/v) sterile glycerol for long term storage at −80 °C.

### Preliminary screening for antigenotoxicity of isolated LAB

#### Bacterial strain

Tester strain *E. coli* PQ37 used for SOS Chromotest was gifted by Prof. Marina Isidori, Department of Environmental, Biological and Pharmaceutical Sciences and Technologies, Second University of Naples, Italy. It was maintained by inoculating nutrient agar plates with 10% ampicillin and incubating at 37 °C for 24 h and preserved at −80 °C.

#### SOS chromotest

Antigenotoxicity of isolated LAB was assessed by SOS Chromotest using DMH and 4-NQO as genotoxicant as described by Quillardet *et al*.^35^. Briefly, the isolated LAB were incubated in MRS broth at 37 °C overnight, cold centrifuged at 4000 g for 10 minutes, washed twice and resuspended at a concentration of 10^9^ CFU/mL. An equal volume (20 µL) of LAB suspension and genotoxicant [either DMH or 4-NQO] were incubated at 37 °C for 2 h, cold centrifuged at 4000 g for 10 minutes and the supernatant collected. *E. coli* PQ37 (600 µL) was added to supernatant of genotoxicant co-incubated with isolated LAB (20 µL). After 2 hr incubation at 37 °C with shaking, β-galactosidase and alkaline phosphatase activities were assayed. Results were expressed in terms of reduction in induction factor (IF).

(IF) = Rt/Ro; where Rt = β-galactosidase activity/alkaline phosphatase activity determined for the test, Ro = β-galactosidase activity/alkaline phosphatase activity of negative control (without genotoxicant).

$${\rm{Inhibition}}\,( \% )=100-({\rm{IF}}1 \mbox{-} \mathrm{IF}0/{\rm{IF}}2 \mbox{-} \mathrm{IF}0)\times 100$$

where IF1 is the induction factor of the test compound, IF2 is the induction factor of positive control (with genotoxicant alone), IF0 the induction factor of the negative control.

#### Secondary screening for the anticancerous activity of isolated LAB *in-vitro*

After successful primary screening selected isolates were assessed for their antiproliferative activity against human colon cancer cells *in-vitro*.

#### Cell lines

Colon cancer cell lines Caco2 and HT-29 were procured from the Cell Repository, National Center for Cell Sciences, Pune, India, and maintained in Minimum Essential Medium and RPMI-1640 supplemented with penicillin 10 U/mL, streptomycin 100 µg/mL and 20% and 10% heat-inactivated FBS respectively, by incubating at 37 °C in a humidified atmosphere with 5% v/v CO_2_ in carbon dioxide incubator.

#### Bacterial strains and culture conditions

The isolated LAB were inoculated in MRS broth and incubated at 37 °C overnight; cold centrifuged at 4000 g and the pellet was collected, washed twice with PBS (pH 7.4) and re-suspended at a concentration of 10^9^ CFU/mL.

#### Preparation of cell-free supernatants

Cell-free supernatant (CFS) of isolated LAB was obtained by cold centrifugation of overnight grown LAB cultures at 4000 g for 10 minutes and filtered through a 0.2 µm membrane filter^36^.

#### Cell proliferation assay

Effect of isolated LAB and its supernatant on Caco2 and HT 29 colon cancer cell lines was assessed by MTT assay^37^. Caco2 and HT 29 cells (2 × 10^4^ cells/mL) were seeded in 96 well tissue culture plates and incubated for 24 h. Thereafter, the media was changed with fresh antibiotic free media and lactobacilli suspension (10^9^ CFU/ml; 20 µL) or CFS of LAB (20 µL) was added followed by incubation at 37 °C for 24 h. 20 µL of MTT (5 mg/mL) was then added to each well and incubated for 4 h. At the end of the incubation period, the medium was removed followed by the addition of DMSO (150 µL) to solubilize the formazan crystals. Absorbance was measured at 570 nm using ELISA reader (TECAN Infinite M200). Results were expressed in terms of % survival of cells after treatment and were calculated as (OD of test/OD of control) × 100. All tests were performed in triplicate and repeated thrice. For viable cells, PBS (pH 7) served as control and for CFS MRS broth served as control.

### Probiotic characterization of selected LAB

#### Acid and bile tolerance

To assess the growth of isolates under acidic condition, MRS broth was adjusted to pH 2.5. Overnight LAB cultures were inoculated to the acidic MRS broth and incubated at 37 °C for 1–2 h. Cultures were sampled hourly for viable cell count. Standard MRS broth (pH 6.5) was used as a control.

MRS broth supplemented with 0.3% and 1% oxgall (B3883, Sigma) (w/v) was prepared to assess bile-salt tolerance. Overnight LAB cultures were inoculated to the modified MRS broth and incubated at 37 °C for 2–4 h. Cultures were sampled at 2 h and 4 h for viable cell count. Standard MRS broth served as control.

#### Cell surface hydrophobicity

The method described by Rosenberg *et al*.^38^ was used to assess bacterial adhesion to hydrocarbons. Briefly, LAB suspension (3 mL) and 1 mL hexadecane or Xylene were mixed by vortexing for 2 min followed by incubation at 37 °C. Samples were withdrawn at 2 h and absorbance was measured at 600 nm. The percentage cell surface hydrophobicity was calculated as per Valeriano *et al*.^39^.

$${\rm{H}} \% =(1-{\rm{A}}1/{\rm{A}}0)\times 100$$

where A0 = absorbance of the control; A1 = absorbance of aqueous phase

#### Identification of the selected isolates

The screened LAB isolates were identified both phenotypically and phylogenetically mainly by colony characteristics, Grams staining, catalase reaction as well as by partial sequencing of 16S rRNA. The standard method for bacterial DNA isolation was used to extract genomic DNA from LAB isolates. 16S rRNA gene was amplified using universal primers (UNI 27F- 5′ AGAGTTTGATCCTGGCTGAG 3′, UNI 1492R- 5′ GGTTACCTTGTTACGACTT 3′). The conditions for PCR were 94 °C for 4 min, 94 °C for 30 secs, 49 °C for 40 secs, 72 °C for 100 secs, and 72 °C for 5 min. Amplicon obtained was purified and sequenced. The sequence obtained was subjected to nucleotide Blast at NCBI database for species-level identification of bacteria. Sequences obtained were submitted to the GenBank with the following accession numbers MH656799, MH656803, and MH889142.

### *In vivo* assessment

Based on the *in vitro* screening, isolates showing maximum antigenotoxicity and antiproliferative activity *in-vitro* further *in-vivo* experiment was performed to assess anticancerous effectiveness of the selected LAB.

### Ethical statement

Care, use and disposal of animals were done as per the guidelines of Institutional Animals Ethical Committee (IAEC), Chandigarh and approved by the committee for the control and supervision on experiments on animals (73/IAEC/18; 9/5/18).

### Animals

Male Sprague Dawley (SD) rats (100–150 g) were procured from the inbred population of Central Animal House, Panjab University after the approval of the present protocol by IAEC, Chandigarh, India.

### Housing and husbandry

The animals were housed in polypropylene cages (n = 3 per cage) with a wire mesh top and a hygienic bed of husk (changed regularly) in a well ventilated, temperature and humidity controlled, rat room with 12 h light/dark cycle. The Animals were acclimatized for 7–10 days and were given water and standard pellet diet (Hindustan Lever Products, Kolkata, India) *ad libitum*.

### Induction of colon carcinogenesis

N,N-Dimethyl hydrazine dihydrochloride (DMH) was prepared in 1 mM EDTA saline and adjusted to pH 7.0 with 1 mM NaOH. Single dose of DMH (20 mg/kg body weight) was given intraperitoneally (i.p.) to animals once a week and the treatment was continued for 6 weeks^11^.

### Preparation of probiotic dose

For experimental inoculation, 18-h old isolated LAB culture was cold centrifuged at 4000 g for 10 min, washed, and suspended in phosphate buffered saline (PBS, pH 7.2) to contain 1 × 10^9^ lactobacilli/0.1 ml^11^.

### Experimental design

Animals were randomly divided into five groups. Each group comprised of 6 animals and treated as follows

- **GROUP I** (Control): Animals received a single dose of 1 mM EDTA saline (pH 7.0) i.p. in a week for six weeks.
- **GROUP II** (DMH): Animals received a single dose of DMH (20 mg/kg body weight) i.p. weekly for six weeks.
- **GROUP III** [*Lactobacillus rhamnosus* MD14+ DMH]**, GROUP IV** [*L. plantarum* GMD+ DMH], **GROUP V** [*P. pentosaceus* GMD17A+ DMH]: Animals belonging to these groups were fed orally with 1 × 10^9^ lactobacilli/0.1 ml daily via orogastric gavage for 1 wk. Thereafter, a single dose of DMH was given i.p. weekly, the treatment was continued for six weeks along with daily oral administration of probiotic.

### Follow up of the animals

During the treatment, body mass, lactobacilli count, fecal pH was monitored weekly. A day before sacrificing the animals, feces of animals was collected, for enzyme (nitroreductase, β- glucosidase and β- glucuronidase) analysis. Animals were sacrificed after six weeks of DMH treatment under anesthesia (ketamine) and cervical dislocation. Aberrant crypt foci count, liver function test and histopathological analysis of the colon was then performed.

### Estimation of body mass and growth rate

The body mass of all animals was recorded weekly on ordinary balance (SD-300, S.D fine chemicals Ltd, Chandigarh, India). The growth rate was calculated as described by Verma and Shukla^11^.

### Fecal lactic acid bacterial count

In order to assess the effect of DMH treatment on the beneficial lactic acid bacterial count in the colon, freshly voided fecal material (0.5 g/rat) from each group was homogenized in normal saline, serially diluted, plated on MRS agar and incubated at 37 °C for 24 h and colony forming units (CFU) were recorded^11^.

### Fecal pH

In order to evaluate the fecal pH, freshly voided fecal material (0.1 g/rat) from each group once in a week was diluted in 2 ml of saline, homogenized with a glass Teflon homogenizer and immediately checked for pH with a pH meter (Deluxe pH meter, model 101E) as per Giovanna *et al*.^40^.

### Enumeration of aberrant crypt foci (ACF)

The entire colon was removed and cut into small sections (2 × 5 cm) and was processed immediately for ACF counts. The sections were stained with 0.2% methylene blue and ACF were counted using a light microscope. The total number of ACF/rat was calculated as the sum of the small, medium and large ACF as described by Bird^41^.

### Fecal enzyme assays

Modified method of Goldin and Gorbach^42^ was used to determine the fecal β-glucuronidase and β-glucosidase enzymes.

### Preparation of fecal homogenate

A day before sacrifice, fresh fecal samples were collected and processed immediately. For β-glucuronidase and β-glucosidase assay, fecal samples were suspended in cold pre-reduced 0.1 M potassium phosphate buffer (pH 7.0) and for nitroreductase assay in cold, pre-reduced 0.2 M Tris- HCl buffer (pH 7.8), homogenized and sonicated for 3 minutes at 4 °C. The samples were cold centrifuged at 500 g for 15 minutes and the enzymes were assayed immediately from the supernatant. The fecal protein concentrations in the supernatant were determined as per Lowry’s *et al*.^43^.

### β- Glucuronidase assay

The reaction mixture (1 ml) consisted of 0.02 M potassium phosphate buffer, 0.1 M EDTA, 1 mM phenolphthalein- β-glucuronide and 0.1 ml of sample. The enzyme reaction was run at 37 °C for 15 minutes at pH 6.8 and stopped by addition of 5 ml 0.2 M glycine buffer (pH 10.4) containing 0.2 M NaCl. Absorbance was taken at 540 nm and the amount of phenolphthalein released was determined by comparison with a standard phenolphthalein curve. The β- glucuronidase activity was expressed as microgram of phenolphthalein formed per hour per milligram of fecal protein.

### Nitroreductase assay

The reaction mixture (200 µl) comprised of 0.08 M Tris-HCl buffer, 0.35 mM m-nitrobenzoic acid, 0.5 mM NADPH and 80 µl of the sample. The enzyme reaction was run at 30 °C for 1 h at pH 7.8 and stopped by addition of 300 µl of 1.2 N HCl. The amount of m-aminobenzoic acid produced was then measured using diazotization reaction and readings were taken at 540 nm. The amount of m-aminobenzoic acid produced was calculated comparing with standard m-aminobenzoic acid and expressed as microgram of m-aminobenzoic acid formed per hour per milligram of fecal protein.

### β- glucosidase assay

The reaction mixture (1 ml) comprised of 0.1 M potassium phosphate buffer, 1 mM nitrophenyl-β-D-glucoside and 0.2 ml of sample. The enzyme reaction was carried out at 37 °C for 1 h at pH 7.4 and stopped by addition of 5 ml of 0.01 M sodium hydroxide. Absorbance was measured at 420 nm. The amount of nitrophenol released was determined by comparison with a standard nitrophenol curve and β-glucosidase activity was expressed as microgram of nitrophenol formed per hour per milligram of fecal protein.

### Liver function test

0.5 ml blood/ rat was collected in a microcentrifuge tube through cardiac puncture and serum was prepared and analyzed for Bilirubin, SGOT, SGPT and Alkaline phosphatase using autoanalyzer, Sysmex XP – 100.

### Histopathological analysis

A part of distal colonic tissue was used for histopathological studies. The formalin-fixed colonic tissue was dehydrated in different grades of alcohol. The tissue was dipped in molten paraffin wax and was cooled quickly to prevent crystallization. Thin sections of tissue were cut, and embedded tissue sections were kept in a water bath at 50 °C to remove the wax. Sections were mounted on separate clean glass microscope slides and were stained with hematoxylin and eosin (H&E) stain and were examined by light microscopy.

### Statistical analysis

Results were statistically analyzed and expressed as mean ± standard deviation (SD). The data was analyzed for normal distribution by Kruskal-Wallis one-way analysis of variance (ANOVA) by using PRISM software. Inter-group variation was assessed by two-way ANOVA by using PRISM software. The statistical significance was defined as p and calculated at p < 0.05.

### Ethics approval and consent to participate

All protocols and procedures related to the sampling, care, and management of animals were reviewed and approved by Institutional Animals Ethical Committee (IAEC), Chandigarh and approved by the committee for the control and supervision on experiments on animals (73/IAEC/18; 9/5/18). All experiments and samplings were carried out in accordance with ethical and biosafety protocols approved by Institutional guidelines.

## Supplementary information

Supplementary Dataset 1
