# Supplementary material for: Isolation, characterization and identification of antigenotoxic and anticancerous indigenous probiotics and their prophylactic potential in experimental colon carcinogenesis
Source: Sci Rep. 2019 Oct 14;9:14769. doi: 10.1038/s41598-019-51361-z (PMC6791833; doi:10.1038/s41598-019-51361-z)
Supplement: Supplementary file 1 — Supplementary Dataset 1 [file 41598_2019_51361_MOESM1_ESM.docx]

**Isolation, characterization and identification of antigenotoxic and anticancerous indigenous probiotics and their prophylactic potential in experimental colon carcinogenesis**

Deepika Chandel^1^, Mridul Sharma^1^, Vibhindika Chawla^1^, Geeta Shukla^1*^ and Naresh Sachdeva^2^

**Supplementary Data**

Supplementary Table 1: Aberrant crypt foci (ACF) count in different group of animals. Values are Mean ± SD, *p<0.05 versus DMH-treated.

| Groups of animals | Control | DMH only | *L .rhamnosus* MD14 + DMH | *L.plantarum* GMD + DMH | *P. pentosaceus* GMD17A + DMH |
| --- | --- | --- | --- | --- | --- |
| ACF count | 0 | 38.25±4.43 | 7.75 ± 2.68^*^ | 13.75 ± 5.21^*^ | 16.25 ± 5.21^*^ |
| ACF reduction  (%) | 0 | ------ | 79.73^*^ | 64.05^*^ | 57.5^*^ |
